# Incidence, prevalence, and outcome of moderate to severe neurotrophic keratopathy in a German tertiary referral center from 2013 to 2017

**DOI:** 10.1007/s00417-021-05535-z

**Published:** 2022-01-06

**Authors:** Mathias Roth, Sebastian Dierse, Jan Alder, Christoph Holtmann, Gerd Geerling

**Affiliations:** Department of Ophthalmology, Heinrich-Heine University Duesseldorf, Moorenstr. 5, 40225 Duesseldorf, Germany

**Keywords:** Cornea, Neurotrophic keratopathy, Incidence, Prevalence, Epidemiology

## Abstract

**Background:**

Neurotrophic keratopathy (NK) is an orphan disease, with an estimated prevalence of 1–5/10,000. No data regarding the incidence exists. The primary aim was to evaluate incidence and prevalence of NK at a tertiary referral center in Germany, and the secondary aim was to analyze demographic parameters, etiology, and clinical features and therapeutic outcomes.

**Methods and material:**

All patients treated for NK with serum eye drops (SED), amnionic membrane transplantation (AMT), or penetrating keratoplasty (PK) in 2013–2017 were identified. Age, sex, etiology of NK, visual acuity, disease stage, treatment, and visual acuity were analyzed. Incidence and prevalence of NK in our hospital and the overall population of the city were calculated.

**Results:**

In 63 eyes of 60 patients (56.7% male; 68 ± 16 years), the most common underlying diseases were herpetic infections (23.8%), neurological causes (19%), and diabetes mellitus (14.3%). The annual incidence of NK in our tertiary referral center ranges between 5/10,000 and 3/10,000, the prevalence between 9/10,00 and 22/10,000. In all patients treated with corneal ulcers, the prevalence was up to 27% (2706/10,000). The incidence in the overall population is estimated at 0.1–0.3/10,000, the prevalence at 0.2–0.5/10,000 to 0.5/10,000.

**Conclusion:**

Based on our assessment, the prevalence of NK in the overall population is lower than estimated before. However, in patients with corneal ulcers, the percentage of NK is comparably high. The disease may still be underdiagnosed due to the variety of underlying disorders and unknown comorbidities. Thus, in cases of therapy-refractive superficial keratopathy or ulcerations, NK should be considered more frequently.
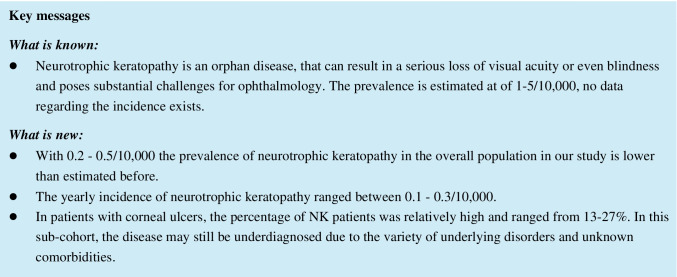

**Supplementary Information:**

The online version contains supplementary material available at 10.1007/s00417-021-05535-z.

## Introduction

Neurotrophic keratopathy (NK) is a corneal condition in which impaired corneal innervation by the ophthalmic branch of the trigeminal nerve leads to reduced corneal healing, epithelial defects, corneal ulceration, melting, and even perforation possibly resulting in a serious loss of visual acuity or even blindness [1, 2].

NK is an orphan disease (ORPHA:137,596, www.orpha.net) and thus epidemiological data on this condition are limited. The estimation of the overall prevalence of 1–5/10,000, respectively 1/2380 in Europe, as stated on www.orpha.net, is mainly based on the prevalence of triggering pathomechanisms such as herpetic infections or iatrogenic damage after neurosurgical intervention [3–5]. Since this estimate does not include other possibly underlying conditions and causes such as e.g. diabetes mellitus or neurodegenerative diseases as multiple sclerosis, the actual prevalence is assumed to be higher [3, 4]. With increasing disease severity, the epithelial and stromal changes in NK are classified in three stages by Dua (see Table 1).

**Table 1:** Clinical findings in the three stages of neurotrophic keratopathy according to Dua [3, 4, 6, 7]

Mild NK/stage I	Moderate NK/stage II	Severe NK/stage III
• Symptoms of dry eye up to severe pain • Reduced tear film breakup time • Increased tear film viscosity • Superficial punctate keratitis and/or “Gaule stains” (spotty epithelial dryness) • Corneal neovascularization • Rose bengal staining of the conjunctiva	• Persistent corneal epithelial defects (smooth or rolled edges) • Corneal stroma edema • Descemet folds • Hypopyon (rare)	• Reduced pain sensation • Corneal ulceration • Corneal perforation • Hypopyon (rare)

The therapy of NK still poses substantial challenges for ophthalmology. Many therapeutic approaches aim to protect the epithelium or to provide it with factors that support the regeneration and restoration of the ocular surface [1, 3]. The focus is on preventing progression, restoring, and subsequently maintaining an intact corneal surface [3]. The therapy of NK is complex, not yet standardized, and adapted according to disease severity.

In the first stage, the disease is hardly distinguishable from dry eye disease and is often treated as such. When the use of lubricant eye drops or gels to promote healing and preserve epithelial integrity as well as therapeutic contact lenses prove to be insufficient, more invasive or elaborate treatment alternatives are required. Commonly employed treatments for moderate to severe NK are the application of serum eye drops (SED), possibly in combination with a contact lens, amniotic membrane transplantation (AMT), emergency corneal grafting (PK), or a combination thereof [3, 8–10].

The aim of this study was to evaluate the incidence, prevalence, and course of disease of NK of patients receiving SED, AMT, or PK at the Department of Ophthalmology of the University Hospital Duesseldorf, a tertiary referral center for severe ocular surface diseases.

## Material and methods

Before initiation of the study, approval was obtained from the Ethics Committee of the Medical Faculty of Duesseldorf (file number 6069R). The study adhered to the tenets of the Helsinki Declaration.

**Fig. 1:**
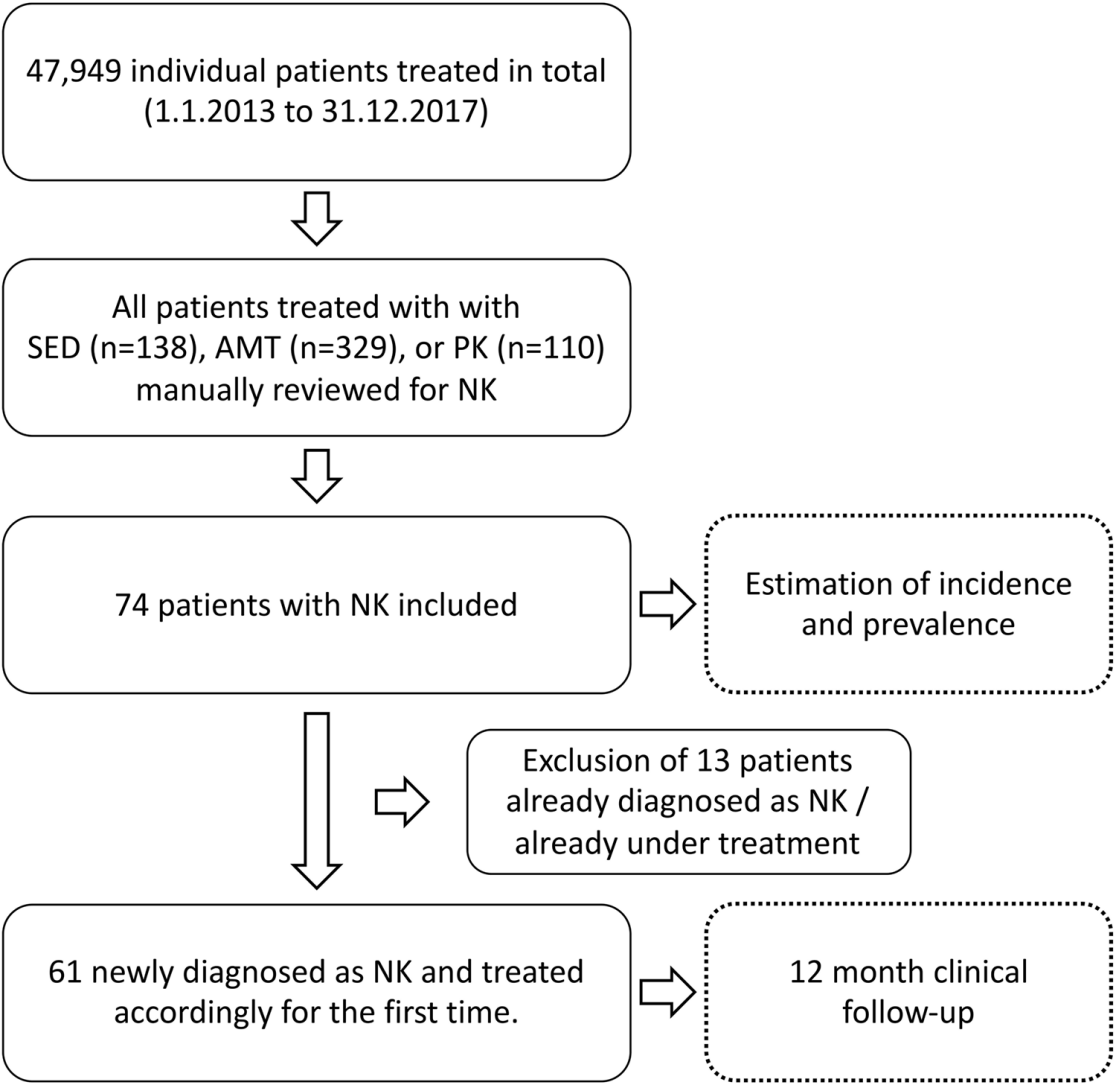
Flowchart of the study inclusion protocol

In the Department of Ophthalmology of the University Hospital Duesseldorf, all patients, which had been treated with SED, AMT, or PK from the 1st of January 2013 to the 31st of December 2017, were counted from in-house registries and the diagnosis was manually reconfirmed from the patient’s records. Inclusion criteria were a documented diagnosis of NK or recorded test result of reduced corneal sensitivity combined with a clinical picture in agreement with NK. Corneal sensation was at least tested with the touch of a cotton thread but in a few cases with Cochet-Bonnet esthesiometer. In the manual review of all records, we identified 74 patients with NK. All other patients were treated for inflammation unrelated to NK or active ocular infection. Those 74 patients were included in the estimation of incidence and prevalence. For the evaluation of the clinical course, only patients with newly diagnosed NK were included. Thus, 13 patients that were already under treatment and/or already were diagnosed NK before 1.1.2013 were excluded for this part of the study (Fig. 1).

### Estimation of incidence and prevalence

To calculate the incidence, all patients were included, which—during the study period—were treated for the first time with SED, AMT, and/or PK (“NK new”). For prevalence, all patients who were treated with SED, AMT, or PK were included, regardless if the treatment episode was for the initial or a recurrent treatment (“NK total”). Incidence and prevalence were calculated for all patients attending the Department of Ophthalmology of the University of Duesseldorf as well as for the subset of inpatients with corneal ulcers, as defined by the diagnosis H16.0 (corneal ulcer) in the ICD-10 (version 2019) in the respective year. Furthermore, incidence and prevalence were estimated for all inhabitants of the city of Duesseldorf. For the calculation of incidence and prevalence in our tertiary center, patient numbers were determined via the clinic’s internal controlling. Patients were counted once per year. Duplicates within 1 year were filtered out. For the estimation of incidence and prevalence in Duesseldorf, epidemiological data was retrieved from the statistical office of North Rhine-Westphalia.

### Clinical evaluation

The clinical records of patients who had been treated with SED, AMT, or PK for a first episode of NK during the years 2013–2017 were retrospectively analyzed. Age, sex, underlying primary disease, previous treatment in another clinic, disease stages immediately before as well as 1, 3, 6, and 12 months after intervention, type and frequency of interventions (SED/AMT/PK), and visual acuity (LogMAR) pre-interventional and post-interventional were recorded. To evaluate differences between interventions, patients who had received only SED (no further intervention method applied), respectively only AMT (no further intervention method applied), were analyzed. As keratoplasties were commonly combined with SED or AMT, the PK group included all patients that had received a PK in combination with SED and/or AMT. Recurrence of NK was defined as a deterioration by at least one NK stage.

### Statistical analysis

Statistical analysis was performed using Prism (Graph Pad Software Inc., version 8.2.1). The data were checked for completeness and plausibility. The normality of distribution of the data was analyzed with the Shapiro–Wilk test. Data is presented descriptively with mean ± standard deviation, and partly with minimum and maximum values. The visual acuity as well as NK stages before treatment and at the end of the follow-up period were compared with non-parametric Wilcoxon matched pairs signed rank test. For group comparisons, Mann–Whitney tests were performed. Spearman’s R was used to investigate correlations. *P*-values ≤ 0.05 were considered statistically significant.

Missing values were replaced as follows: If the value after 1 month was missing, it was replaced by the following monthly value (last observation carried backward). A missing value at a later date was replaced by the values of earlier dates (last observation carried forward). In order to minimize a possible distortion of estimates, we have transferred the value of month 3 when the observation time in month 1 is missing (next observation carried backward). In this case, it is more plausible to assume the value of month 3 after the corresponding intervention, instead of the value of the initial time. In the analysis of NK stage, 12 eyes could not be reliably staged because of incomplete records and were thus excluded for the analysis of NK stage.

## Results

### Prevalence and incidence

In the study period, a total of 47,949 individual patients, respectively 12,418 ± 280 individual patients on average per year, were treated in the Department of Ophthalmology of the University of Duesseldorf. A total of 1849 ± 157 individuals were treated as inpatients and 92 ± 11 were treated for a corneal ulcer (H16.0). During the study period, a total of 74 patients with NK were identified, who had been treated with SED, AMT, or PK during the period of 2013 to 2017. Of those, 61 were newly diagnosed as having NK and treated accordingly for the first time.

Based on the annual total number of patients, the incidence per year in the Department of Ophthalmology of the University of Duesseldorf ranged from 5/10,000 (2013) to 13/10,000 (2016) and the prevalence from 9/10,000 (2013) to 22/10,000 (2016) (Table 2). In the subset of patients with corneal ulcers (H16.0), the percentage of NK patients was relatively high (Table 2). The estimated incidence for the city of Duesseldorf ranged between 0.1/10,000 and 0.3/10,000, the prevalence from 0.2/10,000 to 0.5/10,000 (Table 3).

**Table 2:** Distribution NK new and NK total vs. H16.0 vs. total inpatients vs. total patients overall from 2013 to 2017

A)						
Observation period	*n*					
	NK new	NK total	Inpatient H16.0	Inpatient total	Total	
2013	6	11	84	1574	11,975	
2014	13	19	98	1796	12,714	
2015	13	20	81	1879	12,622	
2016	16	28	110	1993	12,572	
2017	13	23	85	2002	12,207	
B)						
Observation period	%					
	Inpatient H16.0		Inpatient total		Total	
	NK new	NK total	NK new	NK total	NK new	NK total
2013	7%	13%	0.38%	0.70%	0.05%	0.09%
2014	13%	19%	0.72%	1.06%	0.10%	0.15%
2015	16%	25%	0.69%	1.06%	0.10%	0.16%
2016	15%	25%	0.80%	1.40%	0.13%	0.22%
2017	15%	27%	0.65%	1.15%	0.11%	0.19%
C)						
Observation period	NK cases/10,000					
	Inpatient H16.0		Inpatient total		Total	
	NK new	NK total	NK new	NK total	NK new	NK total
2013	714	1310	38	70	5	9
2014	1327	1939	72	106	10	15
2015	1605	2469	69	106	10	16
2016	1455	2545	80	140	13	22
2017	1529	2706	65	115	11	19

A) The absolute numbers of newly diagnosed and/or initially treated NK patients (NK new) and the numbers of all NK patients treated (NK total) are shown. Furthermore, the number of all patients treated because of corneal disease (H16.0) and the number of all patients treated in the Department of Ophthalmology of the University of Duesseldorf (overall) in the respective time period are depicted. B) shows the percentage of NK new and NK total in the three groups: H16.0 vs. total inpatients vs. total patients overall in the study period. The percentage of NK patients in the H16.0 cohort is relatively high. C) For better comparison with other studies, the ratio of NK new and NK total cases per 10,000 in all groups is shown

**Table 3:** Explanation of the approach for the estimation of incidence and prevalence in Duesseldorf

A)			
Year	NK new	NK total	Inpatient H16.0 UKD
2013	6	11	84
2014	13	19	98
2015	13	20	81
2016	16	28	110
2017	13	23	85
B)			
Year	Estimated NK new DUS	Estimated NK total DUS	Inpatient H16.0 DUS
2013	8	14	107
2014	17	25	129
2015	14	22	87
2016	17	30	118
2017	15	27	99
C)			
Year	Inhabitants DUS	Incidence/10,000	Prevalence/10,000
2013	598,686	0.1/10,000	0.2/10,000
2014	604,527	0.3/10,000	0.4/10,000
2015	612,178	0.2/10,000	0.4/10,000
2016	613,230	0.3/10,000	0.5/10,000
2017	617,280	0.2/10,000	0.4/10,000

A) For better clarity, the absolute numbers of NK new, NK total, and inpatients treated because of a corneal ulcer (H16.0) are again shown in this table. B) The number of all patients that were treated as inpatients in Duesseldorf in the corresponding year due to a diagnosis with H16.0 was requested from the statistical office of North Rhine-Westphalia. In those figures, case numbers are counted but not individuals. Based on the data of the hospital controlling, it can be estimated that each patient who had to be treated as an inpatient due to a corneal ulcer was treated on average 1.2 times per year. The figures of the state statistical office regarding inpatients treated because of a corneal ulcer (inpatient H16.0 DUS) were adjusted accordingly. The ratio of patients hospitalized in the Department of Ophthalmology of the University Hospital Duesseldorf, respectively in the whole city of Duesseldorf due to a corneal ulcer, was calculated per year (inpatient H16.0 UKD/inpatient H16.0 DUS). On average between 2013 and 2017, 85% of all inpatients with the diagnosis H16.0 in Duesseldorf were treated in the University Eye Hospital Duesseldorf. According to this ratio, the estimated number of NK patients in the city of Duesseldorf was calculated per year (estimated NK new DUS; estimated NK total DUS). C) Incidence and prevalence per 10,000 inhabitants were calculated based on the total number of residents per year, as retrieved from the statistical office of North Rhine-Westphalia

### Treatment

In 60 of the newly diagnosed patients, follow-up of 1 year was available (56.7% male, mean age 68 years (± 16; min. 13a; max. 94a)). Three of those 60 patients had bilateral disease; thus, we included 63 affected eyes in the analysis of the clinical data. Fifty-one of sixty-three eyes (81.0%) were treated with 84 AMTs, respectively and 19.0% of the eyes did not need an AMT. A total of 58.7% (37/63) had one AMT and 22.2% (14/63) were treated with more than one AMT. Overall, 60.3% (38/63) of the eyes did not need keratoplasty and 32 PKs were performed in 25 of 63 eyes (39.7%). A total of 30.2% (19/63) had one PK and 9.5% (6/63) needed more than one PK. Eighteen of the thirty-two patients with at least one PK also had at least one AMT (56.2%). SED was applied in 15 of 63 eyes (23.8%) for an average treatment duration of 9.7 ± 2.8 months. A total of 39 of 63 eyes (61.9%) had received only one of the three therapeutic options (including recurrent AMTs or PKs). In 20 of 63 eyes (31.7%) two treatment options and in 4 of 63 eyes (6.3%) all three treatment options had been applied simultaneously or at different time points. With no respect to the type of treatment, in 33 of the 63 eyes (52.4%), only a single treatment and in 30 (47.6%) more than one treatment was necessary (mean 2.1 ± 1629 treatments per eye, min. 1 treatment; max 9 treatments).

In 51 eyes, information regarding the epithelial status at the end of the observation period of 12 months was available. A total of 39 of 51 eyes (76%) examined presented with complete epithelial closure. Of the 12 eyes with an epithelial defect at 12-month follow-up, in six (12%), the initial defect was still persisting, while in six (12%), the epithelium had healed but a new defect developed. A total of 12 of 18 eyes (66.6%) receiving only AMT, 17 of 22 eyes (77.3%) receiving PK, and 5 of 5 eyes (100%) receiving only SED had a closed epithelium at the last examination.

### Pathogenesis of NK

In 23.8% (*n* = 15) of eyes with NK, a past episode of herpetic infections (herpes simplex or zoster), in 19% (*n* = 12) neurological causes, in 14.3% (*n* = 9) diabetes mellitus, in 12.7% (*n* = 8) dry eye disease, and in 11.1% (*n* = 7), trauma and microbial keratitis were found as origin of NK. Only in two cases multiple etiologies of the NK could be considered: (1) herpetic eye disease and diabetes and (2) herpetic eye disease and neurological cause (stroke). In both cases, herpetic eye disease was judged to be the primary cause, based on the patient’s history with multiple herpetic recurrences and pronounced postherpetic lesions. In 7.9% (*n* = 5), no underlying disease could be identified (Table 4).

**Table 4:** Distribution of the underlying diseases

Pathogenesis	*n* (eyes)/%
Herpetic infection	15/23.8%
Neurological causes	12/19%
Diabetes mellitus	9/14.3%
Dry eye disease	8/12.7%
Trauma	7/11.1%
Microbial keratitis	7/11.1%
Cause unknown	5/7.9%

Underlying diseases causing NK in 63 eyes of 60 patients. Dry eye disease includes 4 cases of dry eye in connection with rheumatic diseases (Sjögren’s disease (*n* = 2/3.2%), CREST syndrome (*n* = 1/1.6%), rheumatoid arthritis (*n* = 1/1.6%)), 2 cases of idiopathic dry eye disease (3.2%), 1 case with GVHD (1.6%), and 1 case with atopic blepharitis with map-dot-fingerprint dystrophy and keratoconus (1.6%). Trauma cases include history of keratoplasty (*n* = 5/7.9%), corneal trauma (*n* = 1/1.6%), and chemical burn (*n* = 1/1.6%). Microbial keratitis cases include fungal keratitis (*n* = 3/4.8%), bacterial keratitis (*n* = 3/4.8%), and acanthamoeba keratitis (*n* = 1/1.6%). The category *Cause unknown* includes 5 cases in which no definite connection of any underlying general or ocular disease to the NK could be made

### Visual acuity

**Fig. 2:**
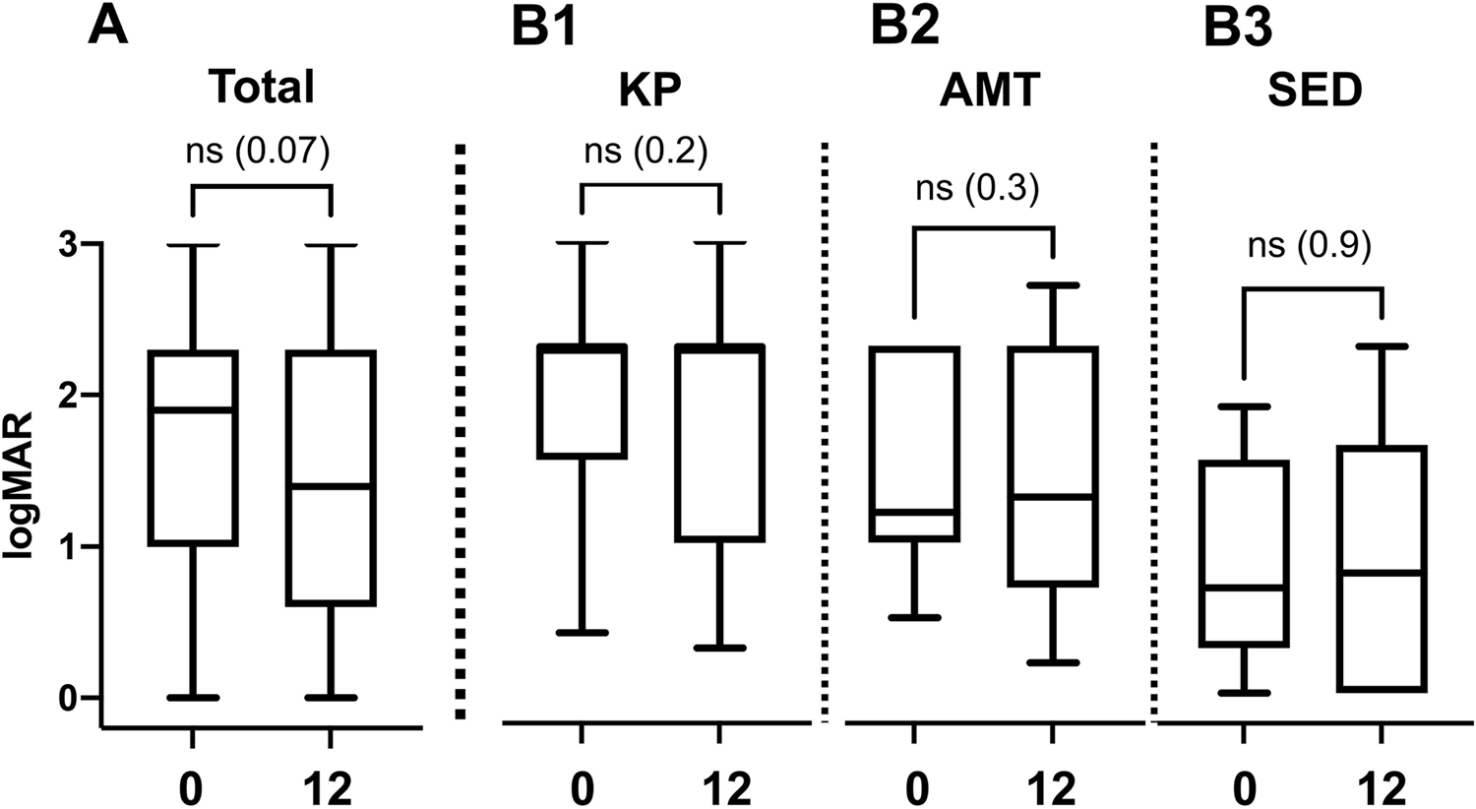
The mean visual acuity (LogMAR) did not change significantly from baseline (1.9 ± 0.77) to 12 months (1.4 ± 0.90) in the entire cohort (A) or in any of the treatment groups (B1-3). ns: not significant

**Fig. 3:**
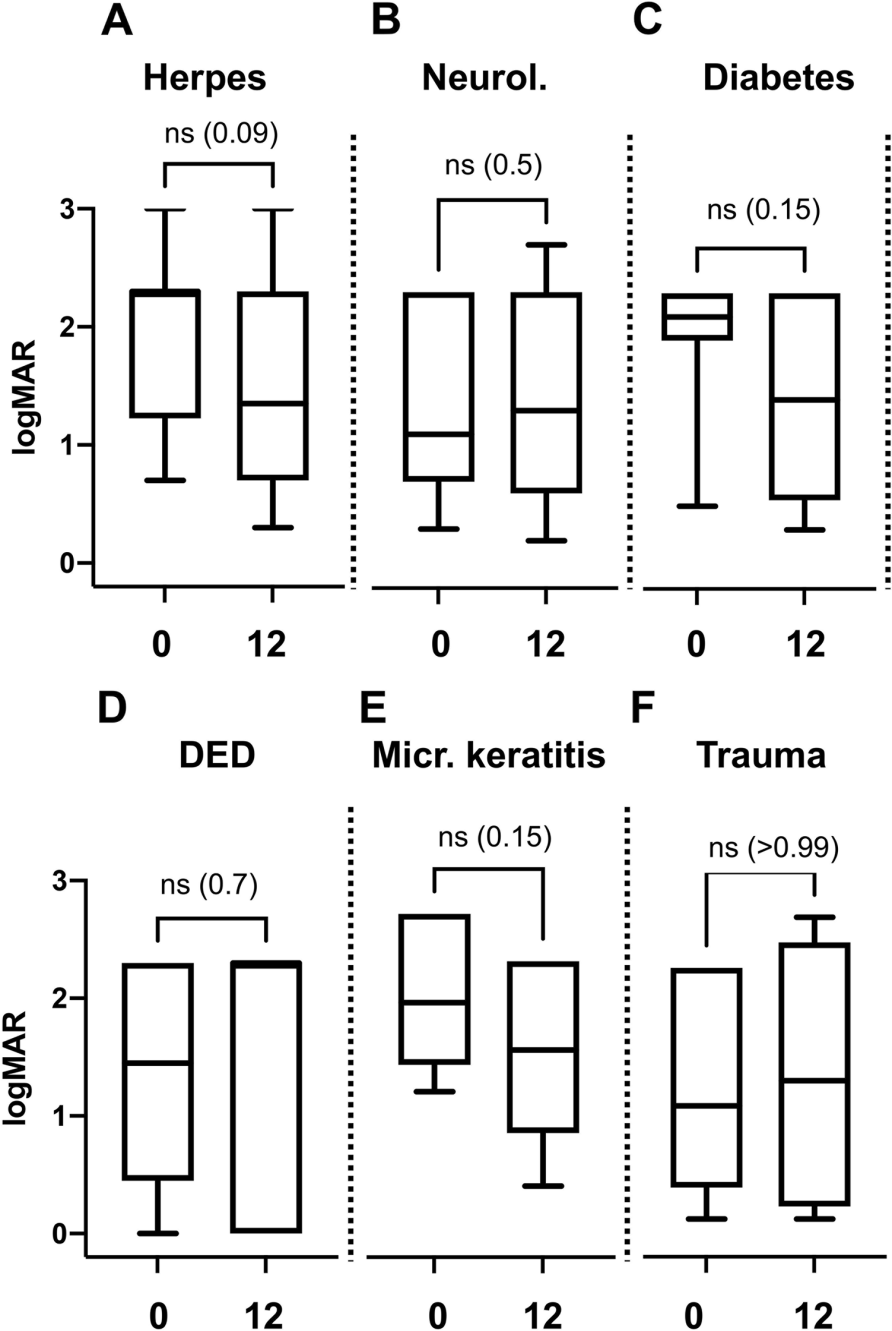
Evaluated by the underlying diseases, there was no significant change of visual acuity from baseline to 12 months in patients with herpes (A), neurological causes (B), diabetes mellitus (C), dry eye disease (DED) (D), microbial keratitis (E), and after trauma (F). ns: not significant

In 49 eyes, sufficient pre-interventional and post-interventional visual acuity data were available. Neither age nor gender had an effect on visual acuity prior to intervention or at the end of the observation period. In the total cohort, as well as in an analysis of the treatment and disease subgroups, visual acuity did not change significantly over time (Figs. 2 and 3).

### Evaluation of NK stages

**Fig. 4:**
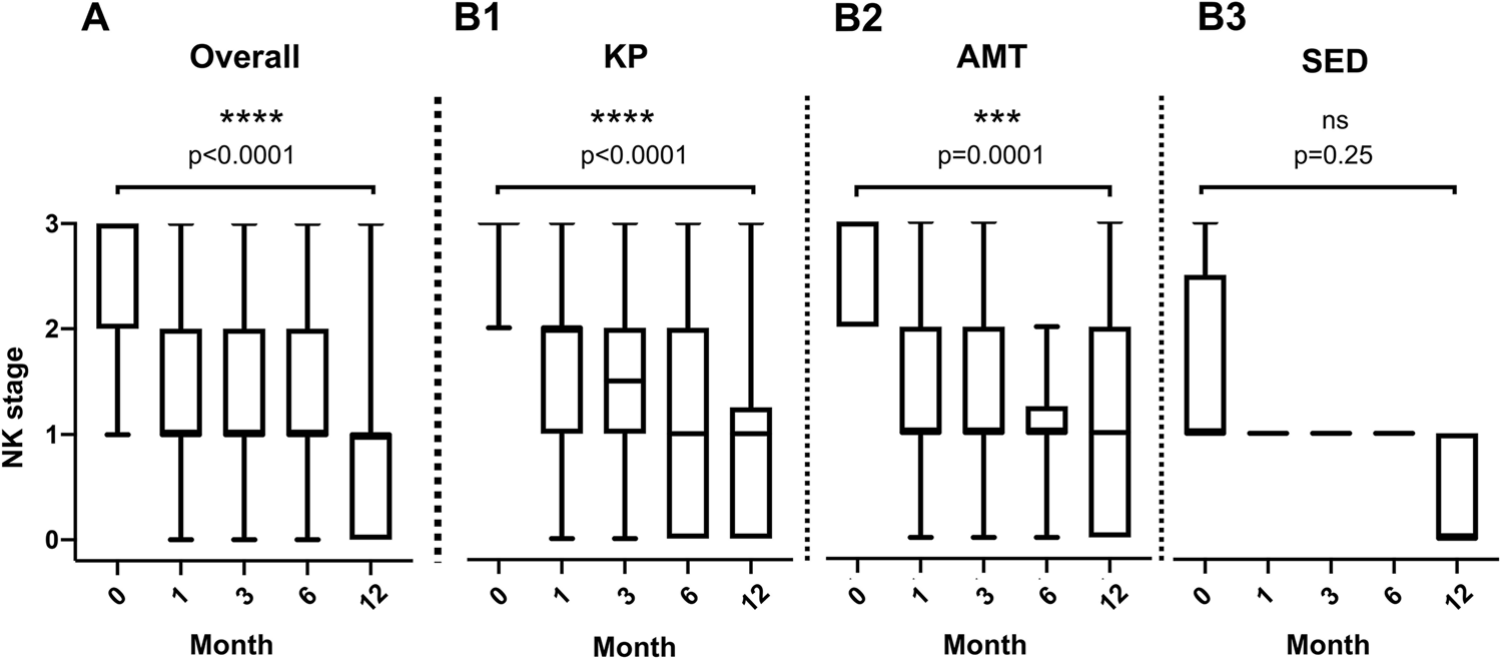
Overall, the NK stage over time improved significantly (*p* < 0.000) (A). Analyzed by the therapy subgroups, there was a significant improvement over time in PK and AMT but not in SED (PK: *p* < 0.0001, AMT: *p* < 0.0001, SED: *p* = 0.0282) (B)

**Fig. 5:**
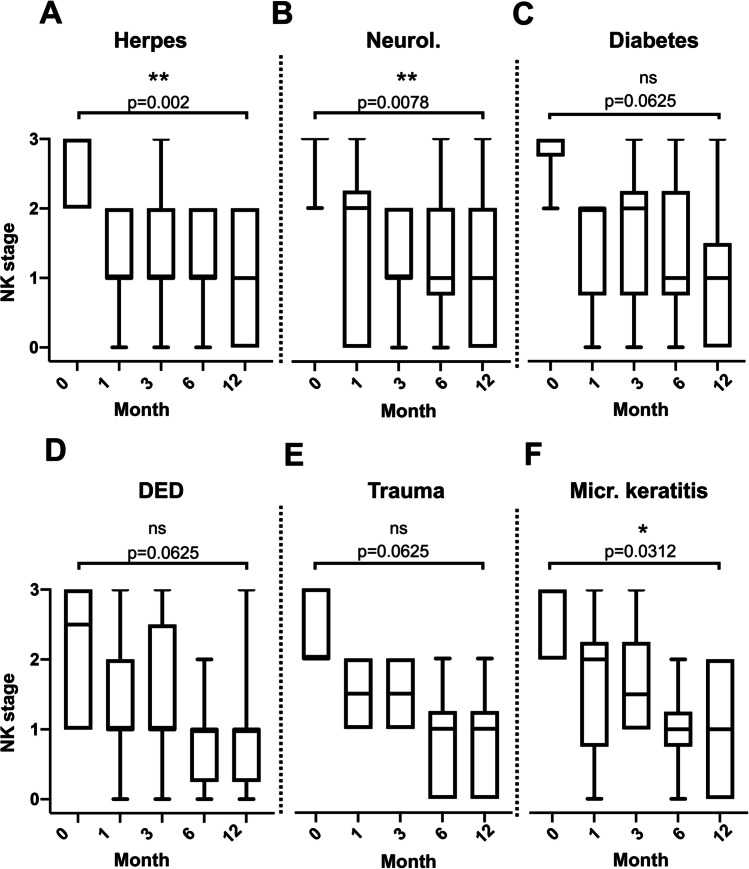
If analyzed by the underlying disease, the NK stages over time (0 vs. 12 months) improved significantly in cases with herpetic infection (A) as well as in underlying neurological disorders (B), and in the cases with history of previous microbial infection (F, but not in diabetes (C), dry eye disease (D), and trauma (E)). Herpes: *p* < 0.0001, neurological causes: *p* = 0.0004, diabetes: *p* = 0.0113, dry eye disease: ns (*p* = 0.0564), trauma: *p* = 0.0164, microbial keratitis: *p* = 0.0067. ns: not significant

Initially, 3 of 63 eyes (4.8%) were in stage 1, 18 of 63 eyes (28.5%) in stage 2, and 42 of 63 eyes (66.7%) in stage 3. The available data for the overall cohort (*n* = 51 eyes) showed a significant improvement of the NK stage in the observation period (0 vs. 12 month; *p* < 0.0001). Initial and final NK stages did not depend on age or gender. Subgroup analysis by treatment method revealed a significant decrease of NK stage for the AMT and PK groups, but not for the SED group (Fig. 4). Analysis of the various subgroups of pathogenesis revealed a significant decrease of the NK stage during the observation period in the cases after herpetic infections, in cases with underlying neurological disorders, and in cases with history of previous microbial infections (Fig. 5). Recurrence or progression at the 12-month follow-up examination was observed in 22.2% of the eyes (14 /63).

For better comparability and clarity, the results regarding applied treatments and outcome have been summarized in Supplementary Table 1.

## Discussion

To the best of our knowledge, so far, no data regarding the incidence of NK and few data regarding its prevalence have been published. Thus, this study on a total of 47,949 patients adds important details about demographics, etiology, and clinical course and therapeutic response in advanced stages of NK in a comparatively large cohort.

Lambiase et al. estimated the overall prevalence of NK to 1.6/10,000, based on the assumption, that NK affects 6% of patients with herpetic keratitis (prevalence: 149/100,000), 12.8% with herpes zoster keratitis (prevalence: 26/100,000), and 2.8% undergoing surgical procedures for trigeminal neuralgia (prevalence: 1.5/10,000) [4]. While our study and Saad et al. included all underlying diseases in the estimation of prevalence, Lambiase et al., due to a lack of available data, could not include NK cases caused by other conditions (e.g., diabetes) [4, 11]. Based on NK-related search words, followed by a manual double-check, Saad et al. found 335 patients (354 NK eyes) among 305,351 patient consultations during an 8-year observation period, equivalent to an NK prevalence of 11/10,000 patients [11]. Whereas our inclusion criteria, based on therapies for advanced stages, lead to a selection of mainly moderate and severe cases (stage 1: 4.8%, stage 2: 28.5%, stage 3: 66.7%), Saad et al. were able to screen also for initial stage NK patients (stage 1: 34.5%, stage 2: 30.5%, stage 3: 32.5%). While the prevalence of NK patients in all patients of our tertiary center is similar to the results of Saad et al., our estimation for the prevalence in the total population of the city of Duesseldorf with 0.2–0.5/10,000 is significantly lower than the estimation of Lambiase et al. But, due to the different approaches, inclusion criteria, and different denominators (number of all patients in a clinic vs. estimation in a total population), it is difficult to compare the published data.

Generally, establishing epidemiological data on rare diseases is a known problem [12]. For example, as described earlier, Lambiase et al. estimate the prevalence of NK in parts on the assumption that NK affects 6% of patients with herpetic keratitis (prevalence: 149/100,000). The prevalence of herpetic keratitis referred to is based on a 3-month survey on the incidence of herpetic keratitis in 412 ophthalmologists in France that was extrapolated to 12 months and the total French population [13]. Thus, the overall NK prevalence of 1.6/10,000, as estimated by Lambiase et al., is based on vague data and sequential calculating steps, each associated with a possible inaccuracy.

Our estimation of incidence and prevalence of NK in Duesseldorf is based on several assumptions and extrapolation too and thus also subject to limitations and caveats. First of all, filter and inclusion criteria lead to a selection of moderate and severe cases only. Furthermore, although H16.239 exists as a specific ICD for neurotrophic keratoconjunctivitis, we could not use this ICD as filter or search parameter, because in Germany, ICDs are used up to the 4th digit only (letter + 3 numbers). All our cases were coded as H16.0. Thus, we assume that NK cases in general are mostly assigned as H16.0, too. Probably the most accurate estimation for NK incidence and prevalence could be achieved through a big data approach, e.g. an analysis of national health insurance data from government or private insurance companies. At present, a research data center that should enable the analysis of government health insurance data is being set up by the German Federal Institute for Drugs and Medical Devices, but is not ready for data mining yet [14]. Nevertheless, we believe that our approach represents a more accurate approximation of the true incidence and prevalence of NK than available studies (see Table 3 for an explanation of our assumptions).

In our cohort of 60 patients, NK was predominantly a disease of an advanced mean age of 68 ± 16 years, which is slightly higher than found by Saad et al. with 63.1 ± 21.0 years in 335 patients [11]. However, there are also isolated cases of younger patients in our cohort (minimum 13 years). In most other studies, the mean age is around 60 years [15–23]. Only Bonini et al. described 43 patients with a comparatively lower mean age (stage 2: 44.0 ± 16.8 years; stage 3: 39.4 ± 19.0 years) with the youngest patient being just 4 years old [24]. As their study focused on the effect of topical nerval growth factor on corneal healing, inclusion and exclusion criteria might explain the difference in age compared to retrospective/real-life data. The proportion of women in our study (43.3%) was similar to the analysis of Saad et al. (50.4%) [11]. In other studies, it ranges from 26.6 to 71.4% [15–19, 22–26]. The reason for this gender disparity remains unclear.

### Etiology

There may be slight differences in the extent of nervous impairment between herpes simplex and herpes zoster [27]. But, as in other studies, in most of our cases, the exact origin could not be specified in retrospective [18, 24]. For this reason, diseases of any herpetic origin were grouped together in our analysis. Herpetic infection is the most common underlying disease in our cohort, which is consistent with several previous reports [11, 17, 18, 20, 21, 26, 28]. However, Chen et al. described a previous keratoplasty as most common cause, Matsumoto et al. and Lee et al. diabetes mellitus, and Bonini et al. chemical burns [16, 19, 23, 24]. As parameters or categories (e.g., of underlying disease) are defined differently in all the studies, they are difficult to compare. Whereas in most studies corneal dystrophies are not mentioned at all, they are the second most common cause in a study by Sanchez-Avila et al. [28]. Saad et al. described the pathogenesis of NK to be as follows: herpetic eye disease in 32.2%, iatrogenic in 31.9%, central nervous system diseases in 27.7%, chronic ocular surface disease in 17.5%, diabetes mellitus in 10.5%, and multifactorial in 34.2% [11]. The high rate of central nervous system diseases was explained by the fact that their hospital was also a referral center for neurologic diseases [11]. Their rate of unknown cause was 8.2% and thus comparable to our cohort [11].

Patients with diabetes mellitus are more likely to develop corneal ulcers [29]. Accordingly, Lookwood et al. proposed that NK must be considered whenever a patient with diabetes mellitus presents with unexplained corneal changes [30]. In our data, diabetes mellitus was the third commonest underlying disease associated with NK (*n* = 9/14.3%).

### Clinical presentation

The clinical presentation of NK can vary substantially [15]. An early stage I is often confused with dry eye disease (DED), possibly delaying the correct diagnosis. In contrast to DED, the rate of bilateral disease in NK is significantly lower and was at 5% in our cohort, which is comparable to Saad et al. (5.7%) and Pflugfelder et al. (6.25%) [11, 18]. In the cohort published by Saad et al., 38% were misdiagnosed initially. The correct diagnosis was established after a mean of 38.8 days, with a maximum delay of 48 days [11]. The rate of misdiagnosis and the duration until correct diagnosis are important parameters and the results of Saad et al. have great relevance for the clinical understanding of the disease. As those parameters were not evaluated in our study, we are not able to add or compare the respective data.

### Therapy

SED, AMT, and PK represent only a part of the complex therapeutic concept, but are commonly used in moderate to severe NK [3]. Tarsorrhaphy can also be considered an important option of NK management [3]. It was not applied in our cohort though. Neither was cenegermin, a recombinant human nerve growth factor (rhNGF), applied as eye drops, as it was not available at that time in Germany.

Although SED, AMT, and PK can prevent progression of the disease and achieve epithelial healing, these options—unlike corneal neurotization—are only symptomatic rather than curative [3, 4, 6, 7, 31]. Thus, around half of the cases in our study needed more than one treatment during the observation period and almost one-third received a combination of at least two different types of treatment.

In the cohorts described by Saad et al. and Hsu et al., almost 70% of the patients were treated sufficiently with medical therapy only including lubrication with artificial tears, ointments, autologous serum, and contact lenses, as well as punctal occlusion, or antibiotics [11, 17]. This is in contrast to our study, where around 92% of the patients were treated also surgically with AMT and/or PK. This can be explained by differences in the inclusion/filtering methods and referral patterns. As NK in early stages is sometimes not distinguishable from dry eye disease and as we do not have in-house registries for contact lens use or tarsorrhaphy, we used interventions (SED, AMT, PK) as filter parameters, thus possibly missing cases of NK stage 1. While only 4.7% (3 of 63 eyes) in our series presented with stage 1 at first examination, in Saad et al., this was 34.5%.

In a recent meta-analysis of the advantages and disadvantages of current treatment options, Di Zazzo et al. found SED to be an effective therapy, although it is associated with time-intensive and resource-intensive individual production and stability issues. They found a healing rate for SED of 81.2 ± 19.2%, and a healing time of 30.6 ± 21.7 days [32]. As in our cohort, they also described the necessity of repetitive surgical interventions in addition to the application of SED [32].

The value of AMT for regeneration of the corneal epithelium is well documented [33–36]. Compared to SED, the healing rate for AMT in our cohort seems to be lower (57.6% ± 21.4; healing time 25.9 ± 11.3 days) [32]. This is likely to reflect a selection bias, as less severe cases are more likely to be started on a non-surgical treatment, such as SED. Turkoglu et al. found no significant difference between SED and AMT in NK, while multilayer amniotic membrane transplantation seems to be more effective than SED in deep postherpetic corneal ulcers [26]. In a randomized, controlled clinical trial, Khokhar et al. treated 15 eyes with NK with AMT comparing it to 15 eyes treated with conventional management (tarsorrhaphy and bandage contact lens). At the end of 3 months of follow-up, 10 of 15 patients (66.67%) in the conventional management group and 11 of 15 patients (73.33%) in the AMT group showed complete epithelialization of corneal ulcers which is not a statistically significant difference [37]. Chen et al. reported that 12 of 16 eyes (15 patients) with NK showed rapid re-epithelialization in a mean of 16.6 ± 9.0 days [19]. We also found AMT to be an effective surgical treatment option for severe neurotrophic corneal ulcers but no long-term follow-up has ever been reported.

Saad et al. report that keratoplasty was performed only in 8 of 344 eyes (2.3%) [11]. Since our patient selection was also based on our in-house corneal graft registry, it included 25 eyes (39.7%) with emergency keratoplasty, representing a possible study bias in inclusion criteria (see above). Also, as patients that were already diagnosed with NK or already under treatment were excluded for the clinical evaluation, comparability is limited.

The relapse rate in our study—defined as a deterioration by at least one NK stage—is high compared to results of a meta-analysis by Di Zazzo et al. (AMT: 1.5% relapse rate, SED, 2.3% relapse rate) [32]. But as the definition of a “relapse” is missing in the referenced publications, a valid comparison is impossible [32].

### Visual acuity

While we found a significant improvement of the condition as a decrease of the NK stages, no significant improvement in visual acuity was found after intervention in our cohort. In contrast, Turkoglu et al. reported an improvement in 18 of 20 (90%) eyes treated with SED and 17 of 22 (77.2%) eyes treated with AMT [26]. Chen et al. describe an improvement of visual acuity in 8 of 16 (50%) eyes treated with AMT [19]. This difference in outcome can be explained by the study bias discussed above. Chen et al. treated severe ulcers, while Turkoglu et al. included mostly patients with corneal erosions and—only to a lesser extent—corneal stromal defects [19, 26]. Saad et al. describe a correlation of initially lower visual acuity with initially higher NK stage, as well as a positive correlation of final with initial visual acuity, age, and initial NK stage [11].

Interestingly, in their study, the time to correct diagnosis did not influence the outcome, which could be explained by the rapid initiation of adequate treatment in many cases [11]. In contrast, Hsu et al. saw little correlation between ulcer grade and visual improvement and no correlation between age and the final visual outcome or visual improvement [17].

## Conclusion

Based on our assessment, the prevalence of moderate to severe NK in the overall population is lower than estimated before. However, in patients with corneal ulcers, the percentage of NK is comparably high. Generally, and also in this subset of patients, the disease may still be underdiagnosed due to the variety of underlying disorders and unknown comorbidities. Thus, in cases of therapy-refractive superficial keratopathy or ulcerations, the evaluation of corneal sensitivity should routinely be implemented, and NK be actively considered more frequently. For the clinical understanding of the disease, the rate of misdiagnosis and duration until correct diagnosis are important parameters that we missed to include in our study. Further evaluation of those parameters needs to be addressed in future studies.

To our knowledge, this is the first study with an estimation of the incidence of NK in the overall population. Despite the limitations due to the retrospective nature of the study and the presumed imprecision, that is inevitable in every approach of estimating incidence and prevalence of a rare disease, in our opinion, this study is of great relevance to the field of neurotrophic keratopathy. Nevertheless, the epidemiology of NK needs to be further investigated in future studies, for example, by analyzing national health insurance data from government or private insurance companies using a Big Data approach. In advanced stages, therapy of NK is complex and rarely leads to a significant improvement of visual acuity. For a better understanding of the disease and in order to further develop more, at best curative therapy options, more data needs to be collected in prospective multi-center studies, including all stages and all available treatment options.

## Supplementary Information

Below is the link to the electronic supplementary material.

Supplementary file1 (DOCX 16 KB)
