# Supplementary material for: Incidence, prevalence, and outcome of moderate to severe neurotrophic keratopathy in a German tertiary referral center from 2013 to 2017
Source: Graefes Arch Clin Exp Ophthalmol. 2022 Jan 6;260(6):1961–73. doi: 10.1007/s00417-021-05535-z (PMC9061695; doi:10.1007/s00417-021-05535-z)
Supplement: Supplementary file 1 — Supplementary file1 (DOCX 16 KB) [file 417_2021_5535_MOESM1_ESM.docx]

| *Supplementary Table 1) Global management data.* | | | |
| --- | --- | --- | --- |
|  |  |  |  |
|  | **n / %** | **Eyes n / %** | **Age** |
| **Patients overall** | 60 / 100% | 63 / 100% | 68 y ± 16 (min. 13y; max. 94y) |
| **Female n (%)** | 26/60 (43.3%) | 28/63 (44.4%) | 71 y ± 14 (min. 32y; max. 94y) |
| **Male n (%)** | 34/60 (56.7%) | 35/63 (55.6%) | 67 y ± 17 (min. 13y; max. 90y) |
|  |  |  |  |
| **NK Stage** | **Stage 1** | **Stage 2** | **Stage 3** |
| **Baseline** | 3/63 (4.8%) | 18/63 (28.5%) | 42/63 (66.7%) |
| **12 months** | 22/51 (43.1%) | 9/51 (17.6%) | 3/51 (5.9%) |
|  |  |  |  |
| **Complete epithelial closure at 12 months** | **Initially stage 1 (n=3)** | **Initially stage 3 (n=18)** | **Initially stage 3 (n=42)** |
| **n (%)** | 3/3 (100%) | 10/18 (55.6%) | 26/42 (61.9%) |
|  |  |  |  |
| **Visual Acuity** |  |  |  |
| **Baseline** | 1.9 ± 0.77 (min. 0; max. 3) |  |  |
| **12 months** | 1.4 ± 0.90 (min. 0; max. 3) |  |  |
|  |  |  |  |
| **Treatment (TT)** |  | **Single TT** | **More than 1 TT** |
| **AMT** | 51/63 (81.0%) | 37/63 (58.7%) | 14/63 (22.2%) |
| **PK** | 25/63 (39.7%) | 19/63 (30.2%) | 6/63 (9.5%) |
| **SED** | 15/63 (23.8%) | -- (Mean duration 9.7 ± 2.8 months) -- | |
|  |  |  |  |
| **Single TT Modality** | 39/63 (61.9%) |  |  |
| **Two TT Modalities** | 20/63 (31.7%) |  |  |
| **All three TT modalities** | 4/63 (6.3%) |  |  |
|  |  |  |  |
|  | **Mean** | **Single TT** | **> 1 TT** |
| **TT No. (independent of modality)** | 2.1 ± 1,629, min. 1; max. 9 | 33/63 (52.4%) | 30/63 (47.6%) |
